# Trends of socioeconomic and geographic inequalities in severe wasting among under-five children in Ethiopia from 2000 to 2019: using the WHO Health Equity Assessment Toolkit

**DOI:** 10.1038/s41598-023-51081-5

**Published:** 2024-01-10

**Authors:** Tsegaw Amare Baykeda, Wubshet Debebe Negash, Tadele Biresaw Belachew, Samrawit Mihret Fetene, Banchlay Addis, Atitegeb Abera Kidie, Alebachew Ferede Zegeye, Tadesse Tarik Tamir, Sisay Maru Wubante, Elsa Awoke Fentie, Desale Bihonegn Asmamaw, Abel Endawkie

**Affiliations:** 1 Department of Health Systems and Policy, Institute of Public Health, College of Medicine and Health Sciences, University of Gondar, Gondar, Ethiopia; 2 School of Public Health, The University of Queensland, Brisbane, Australia; 3 School of Public Health, College of Health Science, Woldia University, Woldia, Ethiopia; 4 Department of Medical Nursing, School of Nursing, College of Medicine and Health Sciences, University of Gondar, Gondar, Ethiopia; 5 Department of Paediatric and Child Health Nursing, School of Nursing, College of Medicine and Health Sciences, University of Gondar, Gondar, Ethiopia; 6 Department of Health Informatics, Institute of Public Health, College of Medicine and Health Sciences, University of Gondar, Gondar, Ethiopia; 7 Department of Reproductive Health, Institute of Public Health, College of Medicine and Health Sciences, University of Gondar, Gondar, Ethiopia; 8 Department of Epidemiology and Biostatistics, School of Public Health, College of Medicine and Health Science, Wollo University, Wollo, Ethiopia

**Keywords:** Health care, Paediatrics

## Abstract

Severe wasting is the deadliest form of wasting caused by a lack of nutritious food and repeated attacks of illness. The World Health Assembly has agreed to reduce severe wasting to less than 5% and 3% by the end of 2025 and 2030. Significant disparities were observed worldwide in progress towards the goal. However, limited evidence of disparity in severe wasting was available in Ethiopia. Therefore, this study aimed to assess trends in socioeconomic and geographic inequalities in severe wasting among under-five children in Ethiopia between 2000 and 2019. The trend in socioeconomic and geographic inequality was assessed using the World Health Organization Health Equity Assessment Toolkit, employing both absolute and relative measures of inequality. Difference (D), ratio (R), slope index inequality (SII), relative concentration index (RCI), and population attributable ratio (PAR) were utilized to assess disparity across wealth, education, residence, and subnational regions. The 95% uncertainty interval (UI) was used to declare the significant change in inequality through time. The proportion of severe wasting increased from 3.8% to 4.7% between 2000 to 2005 and dropped to 2.9% in 2011 to remain constant until 2016. However, the proportion of severe wasting significantly declined to 1.1% in 2019. As indicated by RCI, significant fluctuation in wealth-related inequality was observed in all five survey years but a significant change in wealth-related inequality was observed in 2005 and 2019. Whereas the education-related inequality in RCI of severe wasting steadily increased from −8.8% in 2005 to −24.3% in 2019. And the change was significantly widened from 2011 to 2019. On the other hand, residence-related inequality of severe wasting was observed in 2000 in ratio, difference and PAR summary measures but disappeared in 2019. Between 2000 and 2016, regional inequalities in severe wasting fluctuated between 8.7 in 2005 to 5.9 in 2016 taking the difference as a measure of inequality. Overall, Wealth-related inequality has significantly widened over time with under five children from the richest households being less affected by severe wasting. Education-related inequality was not changed with under five children whose mothers had not attended formal education highly affected by severe wasting. Regional disparity in severe wasting is also exhibited in Ethiopia in all-round surveys with children from Addis Ababa being least affected whereas children from Somalia were highly affected by severe wasting. However, no significant disparity in the type of residence in severe wasting was revealed in Ethiopia. Therefore, special attention should be paid to under-five children living in the poorest households, whose mothers did not attend formal education and children living in Somalia region.

## Introduction

Wasting refers to a condition when a child is very thin for his/her height; which is usually caused by a rapid loss in body weight and/or failure to gain weight that might lead to an increased risk of death if left untreated^1^. Severe wasting is the deadliest form of malnutrition, resulting from insufficient nutritious food and recurrent illnesses such as diarrhoea, measles, and malaria^2^. Weight-for-height nutritional index and mid-upper arm circumference (MUAC) are common ways to measure wasting. Moderate Acute Malnutrition (MAM) is defined by a weight-for-height index between −3 and < −2 Z-score or between 11.5 and < 12.5 cm by MUAC. Severe wasting, also known as severe acute malnutrition, is characterized by a Z score < −3 or MUAC < 11.5 cm^3,4^.

Children ssuffering from severe wasting are susceptible to long-term developmental delays due to weakened immunity and face an increased risk of death^5^. A severely wasted child is 11 times more likely to die of common childhood illnesses than a healthy child^2^. Globally, 13.6 million children under the age of 5 suffer from severe wasting, which is responsible for 1 in 5 deaths of children making severe wasting among the top threats to child survival^2,5^. Although low- and middle-income countries have less than half of the world's under-five children, they account for 75% of all children with wasting^5^. In 2020, 6% of all under-five children in Africa were wasted^5^.

Ethiopia has a high prevalence of wasting, with 7.2% and 1.2% of under-five children moderately and severely wasted in 2019, respectively^6^. On top of that, socioeconomic and area-based disparities in wasting were observed among single studies in the country. For instance, 17.30%, 16.70%, 13.4%, 10.7%, 28.2%, 10% and 11.1% of under-five children were wasted in the east and west Gojjam^7^, north Shewa^8^, Bule Hora^9^, Haramaya^10^, Hawassa^11^, Northwest Ethiopia^12^ and Dilla^13^ respectively.

The World Health Assembly has agreed to reduce severe wasting to less than 5% and 3% by the end of 2025 and 2030, respectively^5^. Ethiopia has notably reduced under-five mortality in the last decades through its multi-sectorial approaches to address malnutrition^14^. Nevertheless, the existence of socioeconomic and area-based inequality could impede the country's progress toward the set goal^15^. Hence, research evidence on identifying the potential inequality in severe wasting will provide evidence to design targeted interventions. However, as per our knowledge, a comprehensive assessment of the trend in socioeconomic and area-based inequality in severe wasting has not yet been conducted in Ethiopia using the World Health Organization (WHO) recommendation. The WHO recommends inequality be measured using absolute and relative measures using both complex and simple summary measures for the selected health indicator to compare the disparities across the inequality dimensions^16^. Hence, employing the WHO-recommended inequality measure would give impactful evidence. Therefore, this study aimed at assessing the trends in socioeconomic and geographic inequalities in severe wasting in Ethiopia for the last two decades using the latest version of the WHO Health Equity Assessment toolkit.

## Methods

### Study settings

With over 120 million population, Ethiopia is the second most highly populated located in East Africa^17,18^. Administratively, Ethiopia is divided into nine regions and two administrative towns. Namely, Tigray, Amhara, Oromia, Southern Nation Nationalities and Peoples Region (SNNPR), Afar, Somalia, Gambela, Benishangul, Harari, Dire Dawa administrative town and Addis Ababa administrative town.

Since the introduction of the second health sector development plan (HSDP II) in 2003, the country has implemented the three-tier health system. Primary health care contains the health posts, health centres and primary hospitals that deliver basic health services. The secondary level contains general hospitals that serve as a centre of referral for primary health care and tertiary healthcare serves the complex and sophisticated health care^19^. Ethiopia has a successful history of reducing maternal and child mortality through its innovative health extension program^20^. However, unacceptable socioeconomic and area-based inequality in basic maternal, and child health and nutrition services exist in Ethiopia^21,22^.

### Data source

This study used the secondary data obtained from the Ethiopian demographic and health survey (EDHS) data as part of the WHO HEAT software 2021 version from 2000 to 2019^23^. The HEAT software was developed to assess inequality in reproductive, maternal, neonatal and child health (RMNCH) services. Hence, 37 health indicators were part of the software with six inequality dimensions such as age, sex, economic status, education, place of residence and subnational region for more than 450 international household surveys conducted in 115 countries between 1991 and 2018. Hence, the software enables us to compare the health indicator’s inequality throughout time and across different countries. The software uses multiple nationally representative data such as Demographic and Health Surveys (DHS), Multiple Indicator Cluster Surveys (MICS) and Reproductive Health Surveys (RHS)^16^.

The EDHS is a regular cross-sectional survey conducted at the community level to represent the overall health status of the country’s population. 2000, 2005, 2011 and 2016 were the major DHS datasets whereas 2019 is the mini-DHS data, that is targeted only at maternal and child health indicators. Two-stage stratification was employed to select households at the country level. Firstly, two enumeration areas (EAs) were created with a proportional allocation depending on the Probability Proportion to Size (PPS). Next, newly created households were selected from the selected EAs systematically with an equal probability after a household listing operation was carried out in all selected enumeration areas. A brief description of each EDHS was presented elsewhere^24–27^.

### Study variable

All children aged less than five in the selected household during the data collection period were included in the study. The outcome variable was severe wasting prevalence in children aged < 5 years which was defined as more than three standard deviations below the median weight-for-height of the WHO Child Growth Standards (yes/no).

### Measure of inequality

The socioeconomic and geographic inequality in severe wasting was measured through different inequality disaggregation and summary measures. We have seen the inequality in severe wasting throughout households’ economic status, place of residence, mother’s educational status, and subnational regions. The economic status was classified in wealth quantile from poorest (quantile 1) to richest (quantile 2) subgroups. The place of residence was classified as urban and rural. The mother’s educational status is no education, primary education, and secondary and above education. The subnational regions were classified into nine regions and two city administrations as listed above. We presented the trend on socioeconomic and geographic inequality in severe wasting using tables and figures and the 95% uncertainty intervals (UIs) were calculated for each subgroup and survey years.

The trend of inequality in severe wasting was analysed using the latest version of WHO’s HEAT software. Based on the WHO recommendation, we have used both relative and absolute measures of inequality that are simple and complex^28,29^. Among the different summary measures incorporated in the software, we have used difference (D), ratio (R), slop index inequality (SII), relative concentration index (RCI) and population attributable risk (PAR) considering the nature of the outcome variable (favourable vs adverse, ordering vs non-ordering), the nature of the data and their more comprehensive application to the inequality assessment^30–32^.

Difference and ratio are simple measures of inequality that do not consider the overall population size to calculate the inequality in severe wasting. Whereas the other three are complex measures of inequality that consider the average population size in calculating the proportion of severe wasting in each disaggregation group. On the other hand, D, SII and PAR are absolute measures of inequality that assess the absolute differences in severe wasting among the subgroups whereas R and RCI are relative measures of inequality that capture proportional differences between subgroups. Hence for ordered dimensions such as economic status and education status, we have calculated all the summary measures whereas, for non-ordered dimensions such as residence and subnational region, we have calculated the inequality using the simple measures of inequality such as D and R together with PAR.

In general, the positive value of the summary measure was indicative of the disproportionately high prevalence of severe wasting among the disadvantageous group such as women who have no education, are poor or live in rural areas whereas the negative values indicate the high prevalence of severe wasting among the advantageous group.

The detailed description of each summary measure was depicted in the technical note of the HEAT software^33^. But to briefly introduce each of the summary measures we have used in this study, D is the simple and absolute measure of inequality calculated as the mean percentage of severe wasting in the one group subtracted from the mean percentage of severe wasting in the other subgroup. Whereas R is the simple and relative measure of inequality calculated as the mean percentage of severe wasting divided by the mean percentage of severe wasting in the other subgroup. The simple measures of inequality were criticized for their ignorance of the middle subgroups and for not considering population size^29,34^.

On the other hand, SII is the complex and absolute measure of inequality that applies to natural ordering subgroups with more than two subgroups like education and wealth. It calculates the difference in estimated values of severe wasting by ranking and subtracting from the most disadvantaged to the most advantaged subgroups using an appropriate regression model taking into consideration all the other subgroups. The positive value shows that severe wasting is more prevalent in disadvantageous subgroups. Besides, RCI is the complex and relative measure of inequality calculated for ordered dimensions that shows the extent to which severe wasting is concentrated among disadvantaged subgroups by considering all population subgroups. Hence positive values indicate a concentration of severe wasting among the advantaged, while negative values indicate a concentration of severe wasting among the disadvantaged group^35^.

Inequality in severe wasting was further summarized with a PAR, which helps to give evidence on the contribution of a group of the population for the overall level change in severe wasting. Hence, PAR would help to know how much the national level severe wasting would be eliminated as the disparity in severe wasting in a certain group is changed maintaining the change in severe wasting the same as the reference population^35^.

### Statistical analysis

The trends of socioeconomic and geographic inequality were disaggregated across the wealth, residence, mother’s educational status and subnational regions for the last two decades in Ethiopia based on the EDHS conducted from 2000 to 2019. A 95% uncertainty interval (UI) was calculated along with the point estimate in each survey year and represented in the table through error bars. Based on the software, inequality of severe wasting is significant if the absolute measure of inequalities (Difference, slope index inequality and population attributable risk) does not include 0 in their 95% uncertainty interval and if the relative measure of inequalities does not contain 1 in their 95% uncertainty interval. Additionally, when examining the trend of inequality between survey years, if the upper limit of the 95% uncertainty interval of one year does not overlap with the lower limit of the 95% uncertainty interval of the subsequent year, it is considered a significant change in the trend of inequality over the years^30–32^. Furthermore, to keep the scientific presentation of the study, we used the Strengthening the Reporting of Observational Studies in Epidemiology (STROBE) guideline^36^.

### Ethical considerations

As the data is publicly available as part of the WHO HEAT software, we have been exempted from providing an ethical clearance. All the ethical requirements were secured by the institution that conducted the survey. Hence, the Institutional Review Board of Ethiopia and the Inner-City Fund International approved the EDHS.

## Results

### Trends of severe wasting throughout equity dimensions

**Figure 1:**
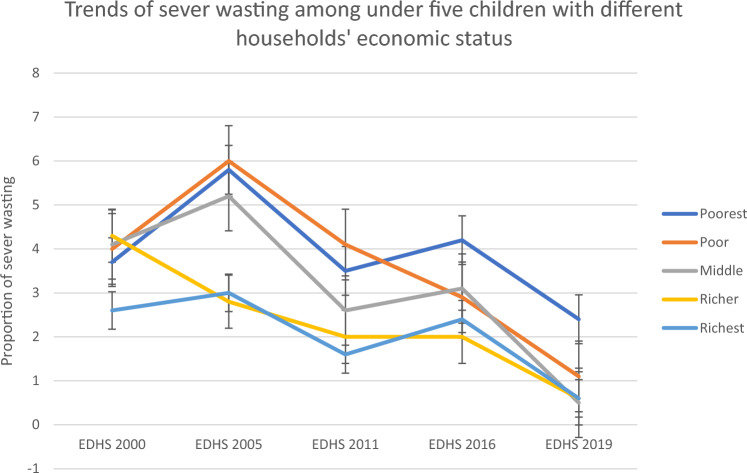
Trends of severe wasting among under-five children with different households' economic status in Ethiopia from 2000 to 2019 (NB: error bar shows the 95% CI).

The proportion of severe wasting across the households’ economic status fluctuated between 2000 and 2019. In all subgroups the worst year was 2005 documenting the highest proportion of severe wasting with 5.8%, 6.0%, 5.2%, 2.8% and 3.0% among the poorest, poorer, middle, richer and richest subgroups respectively. Although a significant decline in the proportion of severe wasting was documented in all economic subgroups between the years 2000 to 2019, a decline in the poorest households was not significant (Fig. 1).

Besides the percentage of severe wasting across mothers’ educational status fluctuated in all survey years taking the peak in 2005 among mothers with no education (5.1%) and mothers with primary education (3.6%) and in 2016 among secondary and above-educated mothers (1.8%). Between the 2005–2019 survey years, the proportion of severe wasting declined significantly among uneducated and primarily educated mothers from 5.1 to 1.7% and 3.6% to 0.6% but not among mothers with secondary and above education.

Similarly, severe wasting significantly dropped from 4.0 to 1.1% among under-five children residing in rural settings but insignificantly declined from 2.0 to 1.0% among urban children between 2000 and 2019. Consistent with the wealth index, the highest proportion of severe wasting was documented in 2005 with 4.8% and 3.2% among rural and urban children, respectively.

**Figure 2:**
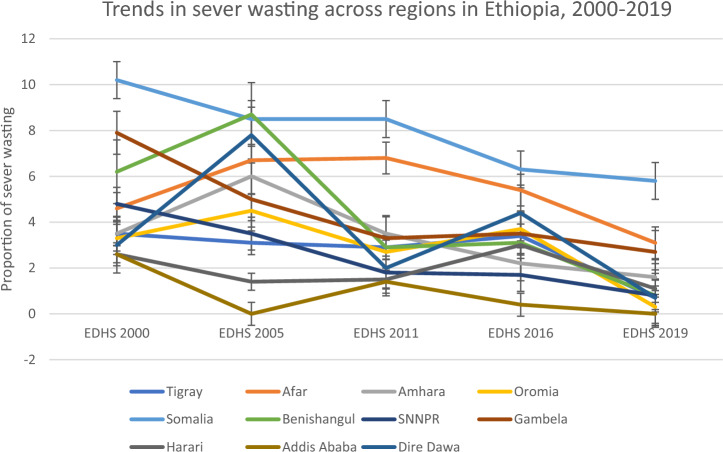
Trends of severe wasting among under-five children in different regions in Ethiopia from 2000 to 2019 (NB: error bar shows the 95% CI).

In Tigray and Somalia regions, the proportion of severe wasting peaked in 2000 at 3.5% and 10.2% subsequently decreasing significantly to 0.7%, and 5.8% in 2019, respectively. In Afar, severe wasting decreased insignificantly from 4.6% in 2000 to 1.9% in 2019 taking the peak in 2011 with 6.8%. In Amhara, Oromia, Benishangul and Dire Dawa subnational regions, the proportion of severe wasting peaked in 2005 at 6.0%, 4.5%, 8.7% and 7.8% respectively but the change through the survey years was significant in Oromia and Benishangul regions but insignificant in Amhara and Dire Dawa regions. But compared to the 2000 survey year, the proportion dropped in all the regions in 2019 to 1.6%, 0.3%, 0.8% and 0.7%, respectively. On the other hand, in SNNPR, Gambela, Harari and Addis Ababa, the proportion of severe wasting steadily declined from 2000 to 2019 from 4.8 to 0.8%, from 7.9 to 2.7%, 2.6% to 1.1% and from 2.6 to 0%, respectively. Among all the regions, the highest (10.2%) proportion of severe wasting was recorded in Somalia region in 2000 and the lowest (0%) proportion was in Addis Ababa in 2005 and 2019 (Fig. 2) (Table 1).

**Table 1:** Severe wasting in under five children in Ethiopia across education, economic status, place of residence and region, 2000–2019.

Inequality dimensions	Category	Survey years														
		2000			2005			2011			2016			2019		
		Estimate	LB	UB	Estimate	LB	UB	Estimate	LB	UB	Estimate	LB	UB	Estimate	LB	UB
Wealth index	Poorest	3.7	2.9	4.9	5.8	4.3	7.9	3.5	2.5	4.9	4.2	3.1	5.6	2.4	1.6	3.6
	Poorer	4.0	3.1	5.2	6.0	4.0	8.8	4.1	2.9	5.7	2.9	2.1	4.2	1.1	0.6	2.2
	Middle	4.1	3.2	5.3	5.2	3.7	7.2	2.6	1.9	3.8	3.1	1.9	4.9	0.5	0.2	1.5
	Richer	4.3	3.3	5.7	2.8	1.6	4.8	2.0	1.1	3.6	2.0	1.3	3.2	0.6	0.1	2.7
	Richest	2.6	1.9	3.5	3.0	1.6	5.5	1.6	0.9	2.6	2.4	1.5	3.8	0.6	0.1	2.5
Mother’s education	No education	NA	NA	NA	5.1	4.1	6.2	2.9	2.3	3.7	3.6	2.9	4.5	1.7	1.2	2.4
	Primary	2.5	1.7	3.7	3.6	2.1	6.0	3.0	2.2	4.1	2.0	1.3	2.9	0.6	0.3	1.5
	Secondary and above	1.2	0.5	3.3	0.1	0.0	0.3	1.2	0.3	4.1	1.8	0.7	1.8	0.4	0.1	1.7
Type of residence	Rural	4.0	3.5	4.6	4.8	3.9	5.8	3.0	2.4	3.7	3.1	2.6	3.8	1.1	0.8	1.6
	Urban	2.0	1.3	3.1	3.2	1.4	7.1	2.1	1.2	3.6	2.1	1.1	3.7	1.0	0.4	2.6
Subnational regions	Tigray	3.5	2.7	4.6	3.1	1.9	5.1	2.9	2.1	3.9	3.4	2.2	5.1	0.7	0.2	2.9
	Afar	4.6	3.0	7.0	6.7	3.8	11.6	6.8	5.5	8.3	5.4	4.1	7.1	3.1	1.9	5.0
	Amhara	3.5	2.7	4.7	6.0	4.3	8.4	3.5	2.4	5.1	2.2	1.4	3.5	1.6	0.7	3.5
	Oromia	3.3	2.6	4.3	4.5	3.2	6.4	2.7	1.8	4.1	3.7	2.8	4.8	0.3	0.1	1.1
	Somalia	10.2	7.6	13.6	8.5	4.6	15.2	8.5	6.0	11.8	6.3	4.0	9.7	5.8	3.9	8.5
	Benishangul	6.2	3.9	9.8	8.7	4.6	15.9	2.9	2.0	4.3	3.1	1.4	6.7	0.8	0.3	1.8
	SNNPR	4.8	3.7	6.2	3.5	2.1	5.7	1.8	1.1	2.8	1.7	1.1	2.7	0.8	0.3	2.3
	Gambela	7.9	5.7	11.0	5.0	2.7	9.2	3.3	1.5	6.9	3.5	2.2	5.6	2.7	1.0	7.4
	Harari	2.6	0.9	7.4	1.4	0.5	4.2	1.5	0.8	2.8	3.0	1.7	5.2	1.1	0.4	3.5
	Addis Ababa	2.6	1.4	4.7	0.0	0.0	0.0	1.4	0.6	3.5	0.4	0.1	1.6	NA	NA	NA
	Dire Dawa	3.0	1.8	4.8	7.8	4.3	13.6	2.0	1.1	3.4	4.4	2.8	6.8	0.7	0.2	2.8

### Inequality in severe wasting by different inequality summary measures

As indicated by RCI, significant fluctuation in wealth-related inequality was observed in all five survey years but a significant change in wealth-related inequality was observed in 2005 and 2019. Whereas the education-related inequality in RCI of severe wasting steadily increased from −8.8% in 2005 to −24.3% in 2019. And the change was significantly widened from 2011 to 2019. On the other hand, residence-related inequality of severe wasting was observed in 2000 in R, D and PAR summary measures but disappeared in 2019. Between 2000 and 2016, regional inequalities in severe wasting fluctuated between 8.7% in 2005 to 5.9% in 2016 taking D as a measure of inequality (Table 2).

**Table 2:** Inequality in severe wasting by inequality summary measures across the various dimensions of inequality, 2000–2019.

Inequality dimensions	Category	Survey years														
		2000			2005			2011			2016			2019		
		Estimate	LB	UB	Estimate	LB	UB	Estimate	LB	UB	Estimate	LB	UB	Estimate	LB	UB
Wealth index	D	1.2	−0.1	2.4	**2.8**	**0.3**	5.4*	**1.9**	**0.5**	3.4*	**1.9**	**0.2**	3.5*	**1.8**	**0.5**	3.1*
	R	1.5	1.0	2.2	1.9	1.0	3.9	2.2	1.2	4.2	1.8	1.0	3.1	4.0	0.9	17.4
	SII	−0.7	−2.0	0.6	**−4.4**	**−6.6**	−2.2*	**−2.8**	**−4.0**	−1.7*	**−2.4**	**−3.6**	−1.2*	**−2.5**	**−3.8**	−1.2*
	RCI	**−3.0**	**−3.3**	−2.6*	**−14.8**	**−17.5**	−12.1**	**−15.6**	**−18.4**	−12.8*	**−12.7**	**−14.9**	−10.5*	**−32.3**	**−43.1**	−21.5**
	PAR	**−1.2**	**−2.0**	−0.5*	**−1.7**	**−2.9**	−0.4*	**−1.3**	**−1.9**	−0.7*	−0.7	−1.4	0.1	−0.5	−1.0	0.0
Mother’s education	D	NA	NA	NA	**5.0**	**3.9**	6.0*	**1.8**	**0.1**	3.4**	1.9	0.0	3.8	**1.3**	**0.5**	2.1*
	R	**2.0**	**1.3**	3.2*	**61.8**	**16.8**	227.3*	2.5	0.7	8.9	2.0	0.8	5.6	4.3	1.0	19.4
	SII	NA	NA	NA	**−6.0**	**−9.9**	−2.0*	−0.5	−1.9	0.9	**−3.9**	**−5.7**	−2.1*	**−2.6**	**−4.4**	−0.9*
	RCI	NA	NA	NA	**−8.8**	**−10.4**	−7.1*	**−1.7**	**−2.1**	−1.4**	**−12.5**	**−14.7**	−10.2**	**−24.3**	**−32.3**	−16.4**
	PAR	NA	NA	NA	−4.5	−9.4	0.4	**−1.7**	**−3.0**	−0.5*	**−1.3**	**−2.4**	−0.2*	**−0.8**	**−1.5**	−0.1*
Type of residence	D	**2.1**	**1.0**	3.1*	1.6	−1.1	4.3	0.9	−0.4	2.3	1.1	−0.3	2.4	0.2	−0.9	1.2
	R	**2.0**	**1.3**	3.2*	1.5	0.7	3.4	1.5	0.8	2.7	1.5	0.8	2.8	1.2	0.4	3.2
	PAR	**−1.8**	**−2.7**	−1.0*	−1.5	−3.3	0.4	−0.8	−1.6	**−0.1**	**−1.0**	−1.8*	−0.1	−0.1	−0.6	0.4
Subnational regions	D	**7.6**	**4.4**	10.7*	**8.7**	**3.7**	13.7*	**7.1**	**4.0**	10.1*	**5.9**	**3.1**	8.6*	NA	NA	NA
	R	**3.9**	**2.1**	7.3*	NA	NA	NA	**6.0**	**2.3**	15.5*	**16.3**	**3.7**	71.5*	NA	NA	NA
	PAR	−1.2	−4.0	1.5	−4.7	NaN	NaN	−1.5	−3.5	0.6	**−2.6**	**−4.3**	−1.0*	NA	NA	NA

*Significant inequality.

**Significant change in inequality with 95% UI.

*D* difference, *R* ratio, *SII* slope index of inequality, *RCI* relative concentration index, *PAR* population attributable risk, *LB* lower bound, *UB* upper bound.

Significant values are in bold.

## Discussion

This study assessed the trends in socioeconomic and geographic inequality in severe wasting in Ethiopia over the last two decades. Overall, the proportion of severe wasting fluctuated over time peaking at 4.7% in 2005 and dropping to 1.1% in 2019. However, a high proportion of severe wasting was concentrated among the poorest subgroups and children whose mothers had not attended formal education. Besides, the regional difference in severe wasting was also observed from 2000 to 2016. But successfully, the residential inequality in severe wasting disappeared in Ethiopia after the 2000 survey year.

Our finding showed that over the last two decades in Ethiopia, the proportion of severe wasting decreased by more than a third from 3.8% in 2000 to 1.1% in 2019. This might be taken as a good track record to achieve the 2025 and 2030 targets to reduce the prevalence of wasting to less than 5% and 3%, respectively^5^. The rate of decline in severe wasting is also higher than in a study conducted in Guinea where the proportion of childhood wasting decreased by less than half from 10.1% in 1999 to 8.1% in 2016^37^. This might be due to the extensive application of maternal and child health services in primary health care units through the health extension program in the health systems of Ethiopia. In Ethiopia, the health extension workers are primarily responsible for monitoring the nutritional, child, and maternal health services provision for households in the frontline. The approach was also the success story of the Ethiopian Millennium Development goal^38^. But the study conducted in Bale zone, Ethiopia showed that the prevalence of severe wasting increased between the years 2014 and 2017 from 3.6 to 4.7%, respectively^39^. It might be due to the difference in study settings and time horizon as the latter is a single site and short period study. but consistent with the study in Bale zone^39^, the highest peak of severe wasting was in 2005 in this study.

Moreover, for the last two decades, wealth-related inequality has been persistent in Ethiopia in favour of the wealthiest households. In 2000, 2005, 2011, 2016 and 2019, the prevalence of severe wasting was concentrated among the poorest subgroups with RCI of −3.0%, −14.8%, −15.6%, −12.7%, and −32.3%, respectively. Besides, the inequality in severe wasting among the poorest and richest subgroups significantly widened in the 2005 and 2019 survey years. In 2005, the prevalence of severe wasting among the richest subgroups was 3% but it was 5.8% among the poorest subgroups the disparity declined in 2019 as the prevalence of severe wasting among the richest subgroups was 1.7% and among poorest subgroups was 2.4%. This can be supported by the study conducted in Ghana where the prevalence of malnutrition was 46.5% in the lowest wealth quintile, but only 8.4% in the highest quintile^40^. The finding indicates the malnutritional problem is still the issue of the poorest^41,42^.

Moreover, education-related inequality was observed in the 2005, 2011, 2016 and 2019 survey years with RCI of −8.8%, −1.7%, −12.5% and −24.3%, respectively. Besides, the education-related inequality in the prevalence of severe wasting has significantly increased since 2011 favouring children whose mothers were highly educated. In 2019, the prevalence of severe wasting was 1.7% among children whose mothers have not attended formal education and 0.4% among children whose mothers have attended secondary and above education. This is in line with the study conducted in Ghana, where mothers with no education account for more than 60% of malnourished^40^.

Furthermore, regional inequality in severe wasting was observed in 2000, 2005, 2011 and 2016 survey years with a difference of 7.6, 8.7, 7.1 and 5.9, respectively. The highest proportion of severe wasting was consecutively observed in Somalia region where 10.2%, 8.5%, 6.3%, and 5.8% of under-five children were severely wasted in 200, 2011, 2016 and 2019 survey years. on the other hand, the lowest proportion of severe wasting was documented consecutively among children living in Addis Ababa, where 2.6%, 0%, 1.4%, 0.4% and 0% of under-five children were severely wasted in 2000, 2005, 2011, 2016 and 2019 survey years. The spatial analysis conducted in Ethiopia also underlined that under-five children living in Somalia region were severely wasted compared to other parts of the country^43^. But throughout time no significant change in inequality of severe wasting was observed among the regions. But this cannot be taken as good progress because the inequality should be narrow throughout time to disappear later. Surprisingly, the residence-related inequality in severe wasting was only observed in Ethiopia in the 2000 survey year. However, after 2000, the disparity in severe wasting among urban–rural residents disappeared in Ethiopia.

Even though the study has employed the national and longitudinal data that represents the high level of sample populations and also observed the trends of inequality based on the WHO recommendation, it has the following limitations. Firstly, the findings of severe wasting are not representative of the current status of the children as the data is generated from the retrospective survey. Secondly, the retrospective nature of the survey might raise issues related to a recall bias. Thirdly, the factors leading to the disparity in severe wasting were not analysed in this study as the study was entirely conducted with the WHO HEAT, which does not allow to conduct a regression analysis. On top of that, the variation in sampling design in the mini-DHS of 2019 and other major DHSs is also one of the limitations of this study. Finally, for some summary measures and inequality dimensions, the toolkit lacks data. Therefore, they filled incomplete and were unable to generate evidence for the specified missed data.

## Conclusion

Overall, the proportion of severe wasting fluctuated over time peaking in 2005 and dropping in 2019. In reverse to the expectation, wealth-related inequality is significantly widened throughout time with under-five children from the richest households being less affected by severe wasting. On top of that, under-five children whose mothers did not attend formal education are highly affected by severe wasting the disparity also appeared in the most recent survey and no significant reduction of severe wasting was made among children with different educational status than their mother. On the other hand, the regional disparity in severe wasting is also exhibited in Ethiopia in all-around surveys favouring the metropolitan cities. Hence, unfairly, children from Addis Ababa were least affected whereas children from Somalia were highly affected by severe wasting. But there no significant disparity in the type of residence in severe wasting was revealed in Ethiopia.

Therefore, the stakeholders in the health systems of Ethiopia should work with a focus on reducing wealth-related inequality, education-related inequality, and regional disparities. Hence, special attention should be paid to under-five children living in the poorest households, whose mothers did not attend formal education and children living in Somalia region.

## Data Availability

The datasets supporting this article’s conclusions are available online as part of the WHO health monitoring database. The DHS data can be acquired online from the DHS database through a formal request by visiting https://dhsprogram.com/ or WHO HEAT software uploaded version at https://whoequity.shinyapps.io/heat/.

## Abbreviations

HEAT: Health Equity Assessment Toolkit
SNNPR: Southern Nations Nationalities and Peoples Region
UI: Uncertainty interval
WHO: World Health Organization
